# Transactions of Societies

**Published:** 1828-04-01

**Authors:** 


### TRANSACTIONS OF SOCIETIES.

### 1. HÆMATEMESIS—MELÆNA.

In a late sitting of the Westminster Medical Society, Mr. North furnished some cases of the above disease, as a subject for discussion. The first case was that of a young woman, who had been in apparent good health, up to the occurrence of the accident, with the exception of a suppression of the menstrual discharge, of a few months' duration. As the vomiting of blood was considered to be vicarious of this suppression; as each discharge seemed to relieve the symptoms of oppression about the præcordia; and, as the patient appeared to bear up well, both mentally and corporeally, against the attack, no apprehensions were entertained by the medical attendants, and a favourable prognosis was given. The patient, however, died suddenly, a few hours after this judgment was delivered. Some dark grumous blood, or secretion of a melænic character, had been discharged by stool. On dissection, the mucous membrane of the stomach and of the upper intestines, was found in a softened state, so that it could be easily scraped off by the scalpel. Beneath, there were numerous points of redness, or of ecchymosis, both in the stomach and bowels. No disease could be recognized in any of the collatitious viscera.

In another case, the symptoms were so nearly similar, that Mr. North did not detail them. Two physicians pronounced the disease devoid of danger—but Mr. North, remembering the foregoing case, gave a doubtful prognosis, notwithstanding that a fellow of the college was against him. The patient died almost immediately after the doctors had decided that she was not to die—at that time! The third case was that of an old woman, and consequently the gastrorhagia was not vicarious. No dissection was permitted. Mr. North remarked that, in his researches among authors on the subject of hæmatemesis, they appeared to him to consider a discharge of blood from the stomach, when vicarious, and when it was not accompanied by severe or threatening symptoms, as always devoid of danger. It was upon this principle that the confident and favourable prognosis was given in the first two cases by the physicians. It was argued, however, by Dr. Johnson, Dr. Barry, and Dr. Copland, that the disease should not be held so cheap—for, in the first place, it might not always be safe to pronounce a vomiting of blood *vicarious*, because a suppression of the menstrual or hæmorrhoidal flux had preceded the accident—and, in the second place, the hæmatemesis is so very frequently the effect of disease in some of the collatitious viscera, as the liver, spleen, &c. that it may be, and too often is, attended with considerable danger. Dr. Johnson, in particular, contended that the disease could not be pronounced devoid of danger, unless it was periodical, and unequivocally vicarious of some other discharge. When it happened, for the first time, no man could tell what might be the event. He observed that a simple and accidental vomiting of blood, unattended with any melænic discharge from the bowels, was infinitely less dangerous than when a black secretion, like tar, constituting melæna, issued per anum. This last affection is always a dangerous disease. Dr. Barry did not agree with Mr. North, as to the general opinion of writers on this subject. Chomel, who had great experience, and who had written, ex professo, on the subject, pronounced hæmatemesis "une maladie grave." In the first case related by Mr. North, he considered the mucous membrane of the stomach and bowels in a state bearing strong analogy to that which sometimes obtains in purpura hæmorrhagica. It was agreed, on all hands, (with one exception,) that the blood issued from the capillary vessels, by a sort of exhalation, and that they were in a kind of atonic condition at the time. The exception to this opinion was enunciated by Dr. Ayre, who believed that the blood, in all these cases, came from the liver, and not from the vessels of the stomach or bowels. This pathology, however, wanted one material point of support—anatomical evidence. Dr. Ayre had not traced the sanguineous or melænic fluid into the ducts of the liver; but he had observed that the premonitory symptoms of hæmatemesis were the same as those which preceded and accompanied other and unequivocal affections of the liver—while the remedy which he had always found effectual in removing the complaint was calomel, the medicine which was also most efficacious in hepatic disorders generally. The worthy Doctor having somewhat inadvertently contemned the pathology of Hippocrates and the ancients, on account of their ignorance of anatomy, and want of opportunities for post-mortem researches, was taken to task by Dr. Barry, who maintained that Dr. Ayre's pathology was founded on precisely the same principles as those of Hippocrates—the symptoms and the effects of remedies. Dr. Ayre had observed a blanched state of the liver and spleen, in some cases where the intestinal hæmorrhage had proved fatal. This, however, was evidently the consequence of the hæmorrhage itself, and would have been the same whether the blood came from the liver or from any other part of the system.

But, whatever might be thought of Dr. Ayre's pathology, (which certainly appeared to us to be much too limited,) the practice which he had found to be so uniformly successful—namely, repeated small doses of calomel, till bile appeared in the motions, and till the melænic discharges ceased, should not be despised. Dr. Copland related some cases which had been treated by the oil of turpentine, both by the mouth and by enema, with complete success. In these cases, there was discharge of half-digested blood from the bowels—but not the melænic stools. Dr. Johnson had seen three cases of idiopathic hæmatemesis, where no melænic discharges were present, and where there was no evidence of organic disease, in which half-drachm doses of sulphate of zinc suddenly arrested the hæmorrhage from the stomach, as soon as vomiting took place. He would therefore be disposed to adopt the same remedy, under similar circumstances—but not otherwise. The combination of melæna with the hæmatemesis would induce him to expect a morbid state, or, at all events, a morbid action in the mucous membrane of the intestines as well as stomach, and consequently a condition not proper for the emetic practice.

Dr. Barry drew the attention of the Society to the superiority of local over general bleeding, in cases of vicarious hæmorrhages. Thus, when hæmatemesis, from suppressed or interrupted menstruation, occurs in this country, we seldom think of attempting to restore the natural or original discharge, by leeching the labia, the hip-bath, &c.—or of applying leeches to the anus, where there has been suppression of the hæmorrhoidal discharge. Yet such is the practice all over the Continent—and such practice is rational. The doctrine of *derivation* (it was observed by Dr. Barry) was now much in disgrace—but perhaps unmeritedly so, as there were few points of ancient pathology better founded in nature or fact.

A gentleman, from the Middlesex Hospital, related three cases of hæmatemesis—two of which proved fatal—the third was recovering. In all of these cases, there was a melænic discharge from the bowels. This gentleman disagreed with Dr. Johnson, in the opinion that the black discharge from the bowels, in hæmatemesis, was an indication of much danger. Dr. Johnson referred this gentleman to a numerical consideration of his own cases, where two out of three died, under this complication.

Upon the whole, it appears to us that there was not sufficient distinction made, by several of the members, between simple vicarious hæmatemesis and that dangerous disease melæna. Although Dr. Ayre did not admit that the disease for which he prescribed calomel was true melæna, (at least so we understood him,) yet we apprehend that it is in this last affection, rather than in simple hæmatemesis, that the above-mentioned medicine is efficacious. At all events, the cases brought forward by Mr. North and others, will render medical men more circumspect in their prognosis, than appears to have been the case in the foregoing instances.

### 2. ERYSIPELAS.

The local treatment of erysipelas by incisions, seems to come more and more into favour. Dr. Dobson, of Greenwich Hospital, has transmitted a paper to Mr. Lawrence (which was lately read at the Medico-Chirurgical Society) in which is detailed the method he has adopted for ten or twelve years past, of making punctures with a lancet, into the erysipelatous parts, whether the erysipelas be cutaneous, phlegmonous, or œdematous. These punctures vary in size and depth—the object being to unload the vessels both of blood and serum. The success attending this practice appears quite astonishing. He employs the remedy in traumatic, as well as idiopathic erysipelas—and also in that red, inflamed, and hardened state of parts which surrounds old ulcers in the extremities. By these incisions, we learn that Dr. Dobson has cured numerous cases of ulcers, that had for years, been rebellious to all other kinds of treatment. Dr. D. conjoined active purgation of calomel and colocynth, with subsequent diaphoretics.

Mr. Earle observed that, however beneficial might be these local means in erysipelas, as adjuvants to constitutional treatment, yet still, in his experience, the disease was, in a majority of instances, connected with, or dependent on, derangement of the general health. On this account, he had found smart purgation, especially with calomel and tartar-emetic, conduce to recovery, in a very remarkable manner. Local means were, of course, to be used at the same time. Dr. Johnson followed on the same side, and contended that, in the majority of cases, erysipelas on the surface was merely a symptom of constitutional disorder. If patients were attentively watched, there would be found preceding phenomena that indicated a derangement of the whole system, as manifested by the tongue, the sensations, the secretions, and the excretions. This being the case, it is pretty certain that cases of erysipelas would, in most instances, get well, by attention to diet and the bowels, with very common local applications.

Mr. Lloyd, since the recent discussions on erysipelas, had had several opportunities of testing the efficacy of the method recommended by Dr. Dobson—small incisions with the lancet—and had experienced the most beneficial results. In one or two cases, where the local inflammation had resisted the enormous venesection of 70 or 80 ounces of blood, at one bleeding, the erysipelas gave way to a series of punctures by the lancet. This treatment had been employed by him in erysipelas of the scalp—the abdomen—and the extremities. Mr. Lloyd never, of course, neglected the constitutional treatment, and always paid great attention to the bowels. Mr. L. confirmed the statements of the author of the paper (Dr. Dobson) as to the good effects of local incisions in the neighbourhood of old and obstinate ulcers. By this auxiliary, he had recently cured an ulcer of the lower extremities, which had resisted all other modes of treatment. Mr. Alcock observed, that this practice was as old as the days of Ambrose Paré, and that Lisfranc always employed it in LA PITIÉ. Mr. Lawrence made some observations on the local treatment of erysipelas, as recommended by Dr. Dobson; and then stated the particulars of a case that had recently occurred, of dissection-wound, in the person of the present very intelligent house-physician of the Fever-Hospital—Dr. Dill. This gentleman, while opening the abdomen of a female who had died of puerperal peritonitis, received a scratch on the back of his hand, from the extremity of a sawn rib. The consequences were, some uneasiness at the time, and a pustule or vesicle the next day, with tense and shining inflammation of the back of the hand, accompanied by great pain. An incision, of about an inch in length, from which a great quantity of blood issued, gave great relief—and this was promoted by the application of leeches, and the immersion of the hand in warm water. Dr. Dill rapidly recovered. Drs. Tweedie and Gregory corroborated this statement. It is probable that, as far as the *instrumental* treatment of erysipelas is concerned, the mode of Dr. Dobson will supersede the long and short incisions by the scalpel. We have reason to know that the lancet cuts heal generally by the first intention, and consequently leave no scar, which might not be the case with scalpel incisions—and, about the eye-lids and face, it is of some consequence to avoid subsequent deformity. We learn that, in some cases, where the eye-lids were greatly swelled with erysipelatous inflammation, the incisions by lancet gave almost immediate relief—enabled the patients to open their eyes,—and brought the erysipelas quickly to a favourable issue.

---

### 3. MEDICAL JURISPRUDENCE.

The following is the recent curriculum promulgated by the College of Surgeons, and to which allusion has been made in the article on medical education in the present number

"*Regulations of the Council relating to the Age and Professional Education of Candidates for the Diploma of the College.*

" I. The only schools of anatomy, and physiology, recognized, are *London, Dublin, Edinburgh, Glasgow,* and *Aberdeen.*

" II. Attendance upon the surgical practice of an Hospital will be recognized, provided such Hospital contain at least one hundred patients.

" III. No person under twenty-two years of age shall be admitted a member of the college.

" IV. The following certificates will be required of candidates for the diploma of this college:

" 1. Of having been engaged six years, at least, in the acquisition of professional knowledge:

" 2. Of having regularly attended three or more winter-courses of anatomy and physiology; and two or more winter-courses of dissections and demonstrations; delivered at subsequent periods.

" *Two courses of anatomy and physiology in Edinburgh or Dublin, which are of six month's duration, and the accompanying courses of dissections and demonstrations will be considered as equivalent to the foregoing attendance.*

" 3. Of having regularly attended two or more courses of lectures on the principles and practice of surgery; one of which shall have been delivered in a recognized school of anatomy.

"4. Of having also attended the following lectures, viz.

"Two courses on the theory and practice of physic of three months each, or one of six months.

"One course on materia medica, and botany.

"Two courses on chemistry, of three months each, or one of six months.

"Two courses on midwifery, of three months each, or one of six months.

"5. And of having attended, during the term of at least one year, the surgical practice of one or more of the following Hospitals; viz. ST. BARTHOLOMEW'S, ST. THOMAS'S, the WESTMINSTER, GUY'S, ST. GEORGE'S, the LONDON, and the MIDDLESEX, in London; the RICHMOND, STEVEN'S and the MEATH, in Dublin; and the ROYAL INFIRMARIES, in Edinburgh, Glasgow, and Aberdeen; or during four years the surgical practice of a recognized provincial Hospital, and six months, at least, the practice of one of the above named Hospitals in the schools of anatomy.

"V. Candidates under the following circumstances, of the required age, and who have been engaged five years in the acquisition of professional knowledge, will be admissible to examination, viz.

"Members, or licentiates in surgery, of any of the legally constituted colleges of surgeons in the united kingdom.

"And graduates in medicine of any of the universities in the united kingdom, provided they have attended lectures, the practice of an Hospital, and performed dissections, as required in regulation IV.

"VI. The required certificates shall express the dates of the commencement and of the termination of attendance on each course of lectures, and dissections; and also of attendance on hospital-practice.

"VII. The required certificates shall be delivered at the college ten days before candidates can be admitted to examination.

"By order,

"EDMUND BELFOUR, Secretary.

"*5th day of January*, 1828."

It is not here necessary to make any comments on the new curriculum, since the whole system is in need of re-construction.* But we may observe, that the College is beginning to act on those principles, which we have so long endeavoured to inculcate—the union of medical with surgical knowledge. We have shewn, on many occasions, the impossibility and the absurdity, of separating the study of surgery from that of physic, and *vice versa*. The College of Surgeons have now broken the ice, and driven their crow-bar through the paper partition that divided them from their brethren in Pall-mall East! They have planted their colours (and very properly too) in the very camp of their rivals. They have only to extend a little the medical flank of their curriculum, in the next edition, and enforce their standing orders by proper musters and inspections (examinations) and then the surgeons of this country, will be, *bona fide*, physicians—or rather they will be, what Hippocrates himself was—GENERAL PRACTITIONERS! The College of Physicians has only to persevere for a few years more in riding on stilts, and the whole practice of the profession will be in the hands of surgeons. Let them carefully avoid all attempts to follow the dictates of reason and common sense—let them continue to prescribe Greek, Latin, and Mathematics, instead of anatomy, surgery, and pathology, and the public, as well as the profession, will soon see the absurdity of consulting those, whose education fits them better for a cloister or a monastery, than for clinical practice. It would be useless for us to offer their high mightinesses any counsel on this occasion. Cardinal Wolsey is their pillar of light—and his PRIEST-CRAFT is their favourite physic.

---

* We shall comment on some items of this curriculum in our next fasciculus.

### HOSPITAL PRACTICE.

### 1. ROYAL INFIRMARY OF EDINBURGH.

GANGRENOUS ERYSIPELAS. AMPUTATION —DEATH.*

We were somewhat startled, on looking at the table of contents of the Lancet, to find the following heading. "Case of gangrenous erysipelas, in which amputation was performed with success." On turning to the case in question, however, all our pleasing anticipations vanished into "air—thin air," for we found that, though the amputation might have been performed *with success,* the patient had most certainly died.

*Case.* D. H. *aged seventy-two,* addicted to drink, was admitted, Jan. 10th, under the care of Dr. Hunter, with gangrenous erysipelas of the arm. The patient appeared to be moribund—the whole limb, up to near the axilla, was enormously swelled, of a dusky red colour, and quite livid over the wrist and metacarpal bones, whilst here and there were several large vesications. He stated that, ten days previously, he had received a bite on the forefinger from a boy, and that nothing had been applied but poultices.

Five or six incisions, about two inches in length each, were made by Dr. Hunter in the arm, and he was ordered wine in small doses, until the pulse rose, with a fermenting poultice to the limb. On the next day, the pulse was stronger, but irregular—tongue coated—bowels confined. *Wine to be continued. Quin. Sulph. gr.* ij. *Mist. camph.* ℥j. *4tis horis.* *12th.* Pulse rather fuller—bowels not yet opened—wounds gaping, and filled with a beer-coloured fluid. *Ol. ricini,* ʒiij. *statim. Camphor emulsion to be omitted—wine—quinine—fermenting poultice.*

We shall not pursue the diurnal details of this case, which "drag their slow length along," with the most tiresome verbosity. On the 13th, the inflammation had extended on the posterior aspect of the arm, but, on the 14th, it was diminishing, and three of the wounds had begun to suppurate. On the 15th, he was no better, but the report states that *a line of separation* was beginning to form *above the elbow,* though it was not a very decided one. On the 16th, it was determined, in consultation, to amputate the arm, which was done, "a little above the middle of the humerus," by Dr. Hunter. The muscles of the fore-arm were flaccid, but not disorganized, and the cellular membrane over the carpus and metacarpus was destroyed, some matter being thrown out amongst the sheaths of the extensor tendons. No mention whatever is made of the state of the stump, or whether the operation was performed in sound or unsound parts! Next day the patient was more comfortable, but somewhat incoherent; tongue moist and cleaner. Quinine to be discontinued, and three ounces of white wine in whey, to be given with barley water and beef tea.

We shall not bore our readers with the prosy details which SCOTUS has given de die in diem; suffice it to say, that no attempt at union took place upon the stump, and that the patient never rallied from the operation. The pulse was never below one hundred—the tongue coated with yellow mucus—the mind more or less affected. On the 20th, he became affected with diarrhœa, which, however, was checked by infusion of roses, with sulphuric acid, and chalk mixture. On the 21st, the pulse was feeble and intermitting, he became affected progressively with rigors, hiccup, &c. and, on the 24th, he died. In the account of the dissection, it is merely stated that the heart was "slightly diseased," and the left lung covered with an old false membrane.

REMARKS. Of this case, reported as it is, we can make, to use a vulgar phrase, "neither top nor tail." First of all we are told that the whole limb was enormously swelled, and of a dusky red colour "from *near the axilla* to the ends of the fingers." On the 13th, the inflammation had "extended on the posterior aspect of the arm," and yet, on the 15th, a *line of separation* was forming, just above the elbow!! Now in our younger days, some twenty years ago, or more, we well remember being taught, that the line of separation was a boundary, formed by a sloughing

* Lancet, No. 232.

process, between sound and unsound parts, between the *living* matter and the *dead*. How, then, we would ask SCOTUS, or any body else, could a line of *separation* form above the elbow, when his own report affirms, that the disease had extended to near the axilla. It would be an odd kind of separation, indeed, which divided the dead matter from the dead!

Secondly, we would ask Dr. Hunter, upon what principle, or for what reason, he made *six incisions* into the limb upon the patient's admission? Did he expect to find matter, or did he expect to prevent its formation? If he did either, we think he did wrong. This was not a case of phlegmonous erysipelas, or of erysipelas phlegmonodes. The tint, we are told, was not bright, but dusky red; the skin was not tense and glistening, but covered with gangrenous phlyctenæ; the pulse did not indicate action, but was "frequent, feeble, and irregular." Was this a case for incisions? We should say it was a case for bark and opium, and that with no sparing hand.

Thirdly, we cannot conceive why the operation was performed about the *middle* of the arm. Surely the object of the surgeon must have been to get fairly *above* the disease, and how this was to be accomplished by amputating in the arm, whilst the erysipelas had extended almost to the axilla, we declare we cannot, for the life of us, comprehend.

Fourthly and lastly, we doubt the propriety of performing the operation at all; for, as far as we can judge, a man of *seventy-two*, who had drunk freely, who was labouring under the most terrible form of erysipelas, and who was in the very worst condition imaginable, either to originate or maintain the reparative process, was no subject for the amputating knife, *however*, or *wherever*, applied.

We know not, for we have no means of knowing, whether this report of Scotus be correct or otherwise; but we will say, that whilst the exact quantities of wine taken, and stools voided, are doled out by the yard, the most important particulars, as the state of the parts in which the operation was performed, the appearance of the face of the stump, &c. are passed over in silence. The consequence of all this is, that the reader gets up from the perusal of the case, just as wise as when he sat down to it.

### 2. MIDDLESEX HOSPITAL.

### MR. BELL ON THE QUESTION OF AMPUTATION.*

The question of primary and secondary amputation, in cases of gun-shot wound or compound fracture, one would imagine to be pretty well set at rest. Mr. Bell, however, has dedicated two clinical lectures to the subject, and, what is more, contrived to make them exceedingly interesting. We notice these observations more for the purpose of commenting on one or two points, than with any intention of detailing them at length.

*Case.* A soldier, æt. 27, whilst drunk, fell in the street, and the wheel of a loaded waggon passed over his leg. He was admitted soon afterwards into the Middlesex Hospital with compound fracture of the leg, the tibia protruding, and simple fracture of the femur in two places, viz. at its centre, and near the trochanter minor. The operation was deferred till next morning, to allow of his recovering from the intoxication, when it was performed high in the thigh. Mr. Bell, being one of those gentlemen who believe it impossible *effectually* to compress the artery at the groin, every precaution was taken against hæmorrhage. *First*, there was the tourniquet applied; *secondly*, there was a gentleman with his thumb on the femoral artery; and, *thirdly*, to keep out the blood from below, there was "a fillet tied firmly round the thigh, below where it was intended to make the incision." No doubt these were proper precautions, and must have been excellent lessons for the pupils of the Middlesex Hospital; but, probably, if the amputation had been performed in the cock-pit, or, as Mr. Bell once expressed it, "on a pack-saddle at the top of the Pyrennees," the surgeon would have contrived to do without *two* of them, at any rate. An incision was then made on the fore part of the thigh, cutting across the main artery, which bled freely. It was accordingly taken up by the tenaculum and tied, as was the profunda. The operation was concluded by making two semicircular flaps, one on the inner, the other on the outer side of the thigh, and removing the bone. The patient hitherto

* Med. Gazette, Nos. 8—10.

has gone on pretty well since the operation.

Upon this case, Mr. Bell makes several remarks. He observes that there are two kinds of compound fracture. A man, for instance, leaps from his gig, and breaks his leg. The limb is twisted, the bone protruding, the case apparently a bad one; "but the surgeon extends the limb, reduces the bone, lays it carefully out upon a pillow, prevents the rising inflammation by cold lotions, or iced water,—and what is the amount of the injury?" The great vessels and different textures are little injured, and the patient will probably do well. If, however, the limb has been crushed, the muscles rent—blood extravasated—the great vessels torn, the appearances to the eye may be the same, but the issue of the case will be very different indeed. The above was an instance of the latter kind of injury, and had not amputation been performed, gangrene, or other fatal termination, would, in all probability, have been the consequence. Passing over Mr. B's reasons for amputating high in the thigh, we come to the question of hæmorrhage. Mr. John Bell, it is well known, denied that the femoral artery could be effectually compressed. It is a different thing, says he, to stop the pulsation, and check the flow of blood. Now, in spite of the authority of Mr. John Bell, surgeons have an idea that they can do both, without any very great difficulty. Sir Astley Cooper, as the story goes, demonstrated this to a young gentleman in the operating theatre of Guy's Hospital, by lifting his thumb from the vessel, and directing the stream of blood, we will not say *upon* him, at pleasure. Mr. C. Bell argues that this practical joke of the worthy Baronet's proves nothing at all; the blood being stopped not by the thumb upon the artery, but by the hands grasping the thigh, and so compressing the collateral vessels. This is ingenious reasoning, but we have seen the circulation completely arrested, without any grasping of the thigh at all, the pressure being made upon the artery by the key-compress.

We contend, indeed, that in the high amputation of the thigh, the tourniquet is at times a positive disadvantage, for it prevents the due retraction of the muscles and integuments, is in the surgeon's way, and instead of arresting hæmorrhage, rather tends to favour its occurrence. This may appear paradoxical, but when we consider that the artery, in this situation, does not lie closely on the bone, but in a kind of hollow between the sartorius and pectineus, we see at once, that by the contraction of these, and the other muscles, the tourniquet is ineffectual in preventing the circulation through the artery, and through the profunda, whilst it is quite, or nearly quite, effectual in preventing its return through the veins. The consequence is obvious—loss of blood. These remarks apply of course, only to the high operation, for the surgeon who makes a boast of never requiring or desiring the tourniquet in any case, is only depriving himself, most unnecessarily, of a very valuable assistant.

With regard to the time of operating in these severe cases of gun-shot wound or severe fracture, Mr. Bell very judiciously observes that, after the patient has once recovered from the shock of the accident, and has revived from the state of depression, "so that he sees by the shattered condition of his limb, that it must be lost, then the operation ought to be performed, and the sooner the better." Mr. B. pays a merited compliment to the surgeons of the British Navy, but singularly enough, omits their brethren of the Army, which is certainly not at all fair to the latter.

The next case which occupies the attention of the lecturer, is one of distorted limb, where the patient wished to have the defective member removed. This wish of a patient's, Mr. Bell thinks, cæteris paribus, should go for nothing in determining the question; as cases have repeatedly occurred, when in compliance with such requests, the limb has been amputated, and the person died. A tailor, for instance, had so crooked a leg, that he could not sit in the proper position on the board. He suffered amputation and died. The case under consideration, was one in which the motions of the knee-joint were much impaired in consequence of an old and ill-set fracture; conjoined with which there were ulcers upon the leg. Mr. Bell taking into consideration the fixed determination of the man to have his leg off, either in that hospital or elsewhere, the utter lameness, and the ulcers which were continually, and would be continually breaking out, at last consented to amputate. Symptoms of tetanus came on, but disappeared; a bad state of the stump supervened, and at the end of five weeks the patient sank.

Mr. Bell makes one observation, in which we do not quite agree with him, namely "that he looked upon the ulcers of the leg as favourable to amputation." Now it certainly seems to us, that these old sores indicate a state of system far from favourable to any great operation, and in one or two cases where amputation was performed for such sores, we have witnessed an unfortunate result.

*Case* 3. A man æt. 54, having received a slight scratch upon his little finger, erysipelas shewed itself, and was relieved by incisions into the arm and hand, giving vent to serum and pus. The erysipelatous inflammation, however, recurred at intervals, an abscess exposed the joint of the thumb, and another surrounds the elbow-joint. In this case, Mr. Bell is adverse to the amputation, because the slight nature of the original injury, and the occurrence and recurrence of the erysipelatous inflammation are evidence of something defective in the constitution, which might, and probably would, equally shew itself in the stump. A young woman in the Middlesex Hospital, was a remarkable instance of this liability to erysipelas. If leeches were applied, an erysipelatous blush extended up the thigh, the same with blisters, caustics, &c. so that the girl nearly died. At last amputation was performed (for disease of the knee-joint) a bad state of the stump was the consequence, ushered in by erysipelatous inflammation, and she narrowly escaped with life.

We have been led farther into these lectures of Mr. Bell's than we intended to have gone, but really the interest of the *matter*, and the engaging *manner* in which it is treated, cannot fail, we think, to be a sufficient apology.

---

### 3. STEEVENS' HOSPITAL, DUBLIN.

#### Vapour-Bath in Tetanus.

Dr. Marsh has published some brief notices of the effects of vapour-bath in this dreadful, and generally fatal malady. The first case was that of a boy, five or six years of age, who was brought into Steevens' Hospital, Dublin, labouring under tetanus. The paroxysms were severe and frequent, and the disease had come on after an injury in the great toe. Calomel in large doses, with other purgative medicines, having failed to act on the bowels—and opium not giving any relief, the vapour-bath was suggested, and the boy was subjected to it, at a heat not above 90 degrees. He remained in the bath six hours. The paroxysm became less violent and less frequent. A third of a drop of croton oil was given every third hour, which operated violently after the fourth dose. Belladonna, and ol. succini, were rubbed along the spine. The bath was daily employed, for several hours each time, and the boy slowly recovered. It was remarked that, on removing the little patient from the vapour-bath, the sore, which had previously been foul and unhealthy, began to improve in appearance, and ultimately assumed a clean granulating aspect.

The next case was also one of traumatic tetanus, but unsuccessful. "The uniform effect of the vapour-bath was to abate the violence of the paroxysms, without, however, influencing, in the slightest degree, the permanent rigidity of the muscles."

In the third case, the patient was an adult, who was under the care of Mr. Cusack, in the aforesaid hospital. Calomel and opium had been given till ptyalism was established; but apparently, without any good effect. The vapour-bath was then tried, and steadily persevered in, till every symptom of the disease gradually gave way. In this and the other cases, the patients were enveloped in a flannel bag, to which, at the lower part, a small tin boiler was attached. Underneath this, a spirit of wine lamp maintained an abundant supply of vapour, the temperature being regulated by a thermometer, introduced into the flannel envelope.

Thus, then, two patients were saved out of three, and one of those saved laboured under traumatic tetanus. We conceive that the long-protracted vapour-bath, at a low temperature, as above described, is infinitely preferable to liquid baths in the common way, or vapour-baths at a high range, where, of course, the stay in the bath must be comparatively short. This plan does not interrupt or interfere with the exhibition of medicine, and we strongly recommend its adoption.

### 4. ST. BARTHOLOMEW'S HOSPITAL.

### INJURY OF THE HEAD—APPLICATION OF THE TREPHINE.

If any one set of cases is more puzzling than another in surgery, it is—"Injuries of the Head." We verily believe that, if a student were to go the round of the different surgical lecturers in this metropolis, he would scarcely find any two agreeing in their practice in these cases. Sir Astley Cooper tells us, that it makes all the difference in the world, whether the cranial fracture be simple or compound. Mr. Travers and Mr. Brodie seem to say that it makes no difference at all! Here, then, upon a mere matter of *experience*, for the comparative danger of one case over another can be known only by experience, sound practical men are at loggerheads. In medicine, alas! there is nothing certain, but death and the doctor's bill.

*Case.* W. B. æt. 30, was thrown from a cart, picked up insensible, and taken to a surgeon, who bled him, and dressed his head. He regained his senses, and vomited frequently, but soon fell again in a state of stupor, and next morning, Jan. 23d, was admitted under the care of Mr. Earle. At this time, he was drowsy —*incoherent*—pupils dilated, but answering to the light—*pulse small and irregular* —extremities cold. Over the upper and lateral part of the left parietal bone, there was a lacerated scalp-wound, but the bone did not appear to be denuded. He was ordered calomel and jalap, and, at 4, P.M. there being some re-action, he was bled. In the evening, 20 ounces of blood were taken from the temporal artery, with some little relief, and, at 12, P.M. was bled again to ℥xvj. Next day he had difficulty of articulation, pulse full and frequent, pupils much dilated. V.S. ad ℥xvj. On the 25th, there was paralysis of the right side of the face, and he passed the urine and fæces involuntarily. *26th.* Partial paralysis of the right side of the body—coma. The wound was sloughy, and the bone beneath bare for about an inch, and, apparently, dead. In consultation, it was determined to apply the trephine. Upon removing the bone, neither blood nor matter was found upon the dura mater, but this membrane had a blueish tint, as if there were blood deposited beneath, and no pulsation of the brain was to be felt. He appeared to recover a slight degree of sensibility after the operation, but soon relapsed. The pupils were greatly dilated—the breathing stertorous—he became violently convulsed, and, at 2, A.M. of the 28th, expired.

*Dissection.* Dura mater not inflamed, but on removing this membrane, a quantity of blood was found effused over the whole cerebrum. The left hemisphere, opposite the point where the bone had been removed, was flattened, by the deposition of a quantity of dark clotted blood, and the posterior surface of its middle lobe was coated with grumous blood. On making a section of this lobe, it was found to have undergone the process of ramollissement to the extent of about two inches. Besides this, a fracture extended from the left temporal bone, through the anterior inferior angle of the parietal, passing across the groove for the meningeal artery. Between the dura mater and bone, at this part, no less than three or four ounces of blood had been extravasated.—*Gazette, No.* 10.

Mr. Earle, according to the report, considered that, in the earlier part of the case, the symptoms of concussion were present, while, latterly, those of compression supervened. On this point, we cannot exactly agree with Mr. E. for, as far as we have seen of concussion, the patient, though drowsy, is not generally incoherent, nor is the pulse "small and irregular." Besides, after having been bled by the surgeon, before his admission into hospital, the patient recovered his senses, but relapsed again immediately. This certainly is not the case with concussion, for, having once recovered from this state, the patient can scarcely be said to fall into it again, but rather to suffer from the inflammatory action, set up as a consequence or sequela of the concussion. It appears to us, that this man laboured under tolerably well-marked symptoms of extravasation throughout, although latterly they were aggravated, partly from the re-action in the system, and partly from the disorganizing process, which terminated in the "ramollissement" of the brain. The practice pursued by Mr. Earle appears to have been highly judicious.*

* This article had been sent to press,

We shall here introduce a case which occurred at St. George's Hospital, and is illustrative of the difficulty which exists in ascertaining the exact nature of the injury which the brain has received.

A boy was brought into the hospital, Aug. 22d, at half past one, P.M. in a state of total insensibility, having fallen from a height of 30 feet. There were two extensive scalp-wounds upon the right side, but the pericranium was entire, and no fracture of the bone could be discovered. The pupils were rather contracted and sluggish—pulse almost natural—surface cool—breathing much like that of a person asleep, and without stertor. When his name was halloo'd loudly in his ear, he was perfectly unconscious of it, but, whilst some bleeding vessels were being secured upon the scalp, he struggled violently, and moaned.

In the evening, he grew restless and fidgetty, and slight re-action came on. He was still insensible, and the pupils were dilated, particularly that of the right eye, but without any strabismus, or convulsive affection, in any of the muscles. V.S. ad ℥viij.

23*d*. Evidently sinking. The surface is cold—the pulse scarcely to be felt—pupils act on the approach of a candle, but the right is still dilated. Towards evening he became rather convulsed, and, at 6, P.M. quietly expired.

*Sectio Cadaveris.* Mr. Brodie, under whose care the patient was, imagined that the symptoms depended upon extravasation beneath the dura mater. On raising the calvarium, no blood whatever was found, either above or below this membrane, and the hemispheres were sliced down to the level of the corpus callosum, without any extravasation making its appearance. In the substance of the cerebrum, a little to the left of the septum lucidum, was a small spot, softened, and containing a little blood. This was also the case with the left optic thalamus, and in the lower part of the posterior lobe of the cerebrum, was a cavity, about the size of an almond, enclosing extravasated blood. A fissure was found running across the optic fossa of the sphenoidal bone, and the liver was of that granular texture found in dram-drinkers.

It would be difficult to say how far the symptoms, in this case, were owing to compression, and how far to concussion. The want of stertor—the coldness of the surface, and the almost natural state of the pulse, would seem to evidence the latter, whilst the unequal dilatation of the pupils, and the general intensity of the symptoms, might rather be referred to the former. These are the mixed cases which occur in practice, and bother the student, who has got by rote all the pretty little distinctions, so *clearly* pointed out in his elementary books.

---

when we received No. 233 of the Lancet, containing a most abusive tirade against Mr. Earle, for the practice which he pursued. There is so large an admixture of slang, "cock-sparrows"—"bats"—"vampires," &c. &c. that, for some time, we could not persuade ourselves but that we were perusing the account of a *mill*, at Moulsey Hurst, between "Barney Aaron" and the "Chelsea Snob," or some such celebrated personages. The sapient critic in the Lancet sports we know not how many notes of admiration, at Mr. Earle's considering the symptoms enumerated above as those of *compression*. Now we have said, and we do maintain it, that the symptoms *were* those of compression, almost from the very commencement of the case, exasperated certainly, at last, by the inflammation going on in the substance of the brain. If the symptoms did not indicate compression, what, in Heaven's name, did they indicate? Concussion? The man in the mask must be even a greater goose than we take him for, if he asserts that difficulty of articulation—paralysis of one side—and involuntary discharge of fæces and urine, are evidences only of concussion. Whether the trephine should have been applied earlier than it actually was, may admit of doubt; but the man who can rate a surgeon, as Mr. Earle is rated, for applying that instrument in a case, where dissection shewed "three or four ounces" of blood *over* the dura mater, and a considerable quantity *under* it, ought to be ashamed of himself.

---

### 5. ST. GEORGE'S HOSPITAL.

#### PECULIAR AFFECTIONS OF THE CRANIAL BONES.

At page 489 of our last Fasciculus we have noticed some observations of Dr. Abercrombie's and Mr. O'Halloran's upon this head. Two cases have lately occurred at St. George's Hospital, under the care of Mr. Rose, which might well have been appended to the article in question.

*Case* 1.* A woman was admitted for bronchocele, but there was *casually* observed a tumour on the head. It had been punctured three weeks previously, and into the puncture a probe was introduced, and vent given to some glairy serum. Next morning rigors and severe constitutional disturbance came on, an erysipelatous blush appeared upon the neck, and on the 3d day she died. On dissection the tumour was found to be of malignant structure, and to pass through a circular opening in the parietal bone, and take attachment (though not a firm one) to the dura mater beneath. This membrane was quite sound, and there was no reason to believe that the tumour had originated from it.

*Case* 2. This patient we had an opportunity of seeing ourselves, and the particulars, we believe, have not been published elsewhere.

Caroline Hollyoake, æt. 20, was admitted Nov. 7th, with extensive disease of the right parietal bone. The outer table was completely exposed for a space about the diameter of a crown-piece, but longer, and the edges of the scalp around were curiously tucked in. The bone was rough and blackened, and around it a line of separation had been commenced, but was ineffectual in checking the disease, for the probe passed beneath the scalp, and discovered dead bone for some distance around. The coronal suture crossed the exposed bone in front, and between its serrated edges, which were more apart than they should be, pus was seen undulating in correspondence with the pulsations of the brain. The menses had always been irregular, and for the last three years had ceased entirely. About a year previously she began to be affected with pain in the head, and hysteria, and she first noticed matter amongst the hair, nine months prior to admission. An abscess appears to have formed, and the part of the scalp to have been destroyed by ulceration, Besides this disease of the cranium, there was likewise dead bone in the lower jaw. There was no reason whatever to suspect a syphilitic taint—she had never received any blow upon the part—nor could she account for the affection in any way. On the 13th, Mr. Rose removed a portion of the blackened outer table by an elevator, and on the 29th he divided the scalp, and removed a still larger portion by means of Hey's saw. Large flabby granulations were exposed beneath, which pulsated strongly; but whether they arose merely from the diploë, from the dura mater, or partly from one and partly from the other, it would be difficult to say. The patient was going on pretty well—the pulsation had distinctly lessened, and her health was certainly not suffering much, when she was persuaded by her stupid friends to leave the hospital, lest she should be made to undergo any more operations.

At this time the probe passed for some distance across the os frontis, and there was altogether a very large amount of diseased bone on the right side of the head. What was the cause of all this it would be difficult to say, though it must be owned that the girl was of a puny, scrophulous habit of body. It is curious how little disturbance these affections cause to the sensorium. In the first case, the patient was scarcely aware of the existence of the tumour, and, in the second, there was no paralysis, no affection of the mind whatever. Mr. O'Halloran objects to the use of the trephine, but upon what grounds we know not. If there is a mass of diseased bone in the skull, particularly if the outer table only is affected, it surely must be an object to get rid, as quickly as may be, of this dead and irritating body. If, indeed, there be symptoms of irritation to the brain, as convulsions, paralysis, epilepsy, &c, then we conceive that the surgeon, cæteris paribus, has no choice in the matter, but is imperatively called upon to remove the affected bone.

---

* Lond. Med. Gazette, No. 1.

### 6. ST. THOMAS'S HOSPITAL.

### On Non-union of Fractured Bone. By Mr. Amesbury.

A very interesting paper upon this subject was read before the Medical Society, at Bolt-court, on Monday night, by Mr, Amesbury. He detailed at length, a case, which had occurred under the care of Mr. Green at St. Thomas's Hospital, in which several methods of treatment had been unsuccessfully employed, and the patient ultimately had the limb amputated.

A strong, healthy sailor, æt. 36, was admitted March 11th, 1827, with fracture of the thigh about its middle-third, which had existed for twenty-four weeks, and been treated in a very ineffectual manner at a Portuguese hospital. The fracture was oblique, very loose, and the lower portion of the bone was drawn up along the inner side of the upper one, to the extent of two inches and a half, but extension restored the limb to its natural length. By the politeness of Mr. Green, Mr. Amesbury was allowed to apply his apparatus, so as "to retain the limb of its proper length, and press the fractured surfaces strongly together." Much pain followed the application of the apparatus, and continued more or less for ten weeks, when it was removed, but no union had taken place. A seton was next passed between the ends of the broken bone, extension being at the same time kept up by fixing the foot and the pelvis, and applying a splint along the outer side of the limb. On the 19th day after its introduction, it was necessary to remove the silk, in consequence of matter having burrowed beneath the fascia, and affected the constitution somewhat severely. Thickening of the soft parts had taken place, but nothing more. During the suppuration which followed, pressure and extension by means of splints were assiduously kept up, but without effect, and at the expiration of nine weeks from the withdrawal of the seton, Mr. Green proceeded to cut down upon the ends of the bone. A semi-circular incision was made in the front and outer side of the thigh, through part of the rectus and vastus externus, and the bone exposed, the ends of which were found to be connected by a dense capsule. This was removed from the upper portion of the bone, but in consequence of the under portion being drawn up on the inner side of the former, it was found impossible to get at the capsule connected with it. Half an inch of the upper portion of the bone was sawn off, and discovered to be soft and spongy from interstitial absorption.

After a time the splints were again very carefully applied, but at the expiration of nine weeks, no union having taken place, the limb was amputated at the poor fellow's own request. On examination of the bone, the capsule was found to be again complete, the greater part of it being as thick as the capsule of the hip-joint, and its inner surface smooth like a synovial membrane. The ends of the bone were rounded, and where they came in contact, flattened and covered with a fibro-cartilaginous membrane resembling the intervertebral substance.

Upon this case Mr. Amesbury made some very apposite remarks. In 41 cases out of 45 which he had witnessed, the non-union was attributable, in his opinion, as in the one detailed, not to weakness of constitution, but to imperfect adaptation of mechanical means. In eighteen cases, Mr. A's plan has been successful, most of them having existed ununited above six months, one nine, one ten, one eleven, and two fourteen months before coming under his care. In two cases only, this being one,* has Mr. Amesbury failed. In all *very loose* fractures of long standing, Mr. A. would be disposed, after an unsuccessful trial of pressure and rest by proper apparatus, to cut down upon the ends of the bone, remove by the knife the new capsule, and stimulate the ends of the bone either by a wash, or by the application of a caustic. This, with the subsequent assistance of due mechanical support, Mr. A. considered as affording the best chance of cure in these very troublesome cases.

---

* The other, if we are rightly informed, occurred at St. George's Hospital. We shall take an early opportunity of giving the particulars.

No. 1. Mr. Lawrence to Dr. Johnson.

18, *Whitehall-place*,
13*th Feb.* 1828.

Sir,—A friend of mine, who reads your periodical publication, has sent me the two last Fasciculi, calling my attention to two statements which they contain respecting myself. As you address to me, in one of these, a kind of challenge either to admit or deny it, and as both are contrary to fact, I think it necessary to notice them; and I beg that you will insert this letter in your next Number, that my contradiction may reach those who have seen your erroneous assertions.

In your comments on the case of Mrs. Denmark, you say "we have been informed, on the very best authority, that on the very day after the operation, and afterwards on the 10th or 12th day, consequently before the ligature came away, the pulse was distinctly felt by Mr. Lawrence and others in the right arm. If this information be incorrect, Mr. Lawrence can easily contradict the statement, when we will give our authority, who also felt the pulse."—*Fascic.* II. p. 469.

I did not see Mrs. Denmark on the day after the operation, nor within a week of that time. I have seen her only once since she was operated on, namely, about the 10th day. I then visited her with Dr. Tweedie, at the invitation and in presence of Mr. Wardrop, of whom she was a private patient. I do not remember the details of her symptoms at that time, having no inducement to make minutes of a case under the care of another gentleman: but the impression on my mind is, that a feeble pulsation could be felt in the right radial artery. I am not certain whether I ever saw Mrs. D. before the operation; if I did, it was without having the opportunity of inquiring minutely into her case, or forming an opinion respecting the treatment that it required.

In describing the case of J. Nowlan, you say "on the 13th day he was better, but the eye-ball was found to be greatly protruding, which was attributed, by Messrs. Lawrence and Wardrop, to a powerful compression on the brain, exercised by an aneurismatic state of the vessels of the dura mater, communicating through the skull with the tumour on the head.—*Fascic.* III. p. 500.

I never entertained nor expressed any such opinion.

Having thus taken the trouble of contradicting two mis-statements, because they involve others as well as myself, I beg that your readers will not consider that I admit the correctness of those representations, which I leave uncontradicted. Your report of the proceedings at the Medical and Chirurgical Society, contained in the same Fasciculus, which ascribes to me the absurdity respecting the case of J. Nowlan, would alone render it necessary for me to enter this protest. I shall make no remark either on the correctness of that report, or on the kind of taste and feeling which it displays. The former will be best estimated by those who were present at the meeting, while I doubt not that the latter will be properly appreciated by your readers generally.

I remain, Sir,
Your obedient servant,
William Lawrence.

Dr. James Johnson.

No. 2. REPLY.

Let us now see what foundation there is for all this supercilious and taunting exposure of supposed mis-statements.

First. In respect to Mrs. Denmark's case, the pulse *was* felt in the right arm *on the very day after* the operation, by Dr. Barry and others, (who have made no secret of it)—on the 10th day, by Mr. Lawrence himself, according to his own admission. The facts, therefore, respecting the pulse, are strictly correct—though by not exactly assigning to the observers the precise dates of their respective observations, we have made Mr. Lawrence (in the place of "*others*") appear on the second, instead of the tenth day after the operation. We say again, the *facts respecting the pulse are strictly correct;* and the whole of this mighty error consists in putting "*Mr. Lawrence*" before "*others*," in the statement. We give Mr. L. all the glory he can claim, for the detection of such "erroneous assertions."

Secondly. Mr. Lawrence evidently thinks he has branded *us*, beyond redemption, with the stigma of mendacity, in the case of J. Nowlan. Let us see what the "invaluable journal," the Koran of the party, says upon this occasion.

In the Report from the HOSPITAL OF SURGERY, (Lancet, No. 214, p. 24-5,) we find the following passages :—

"On the thirteenth day, the stupor and delirium had subsided, but the blindness was undiminished, *and the eyeball was found to be protruding from the orbit*, with œdematous effusion under the conjunctiva, and in the palpebræ.

"These singular and distressing symptoms (including, of course, the *protrusion of the eye-ball—Ed. of Med. Chir. Review*) gave rise to much speculation, as to the cause of their occurrence. Some *physiologists* ascribing them to a want of sensibility in the brain and its nerves, owing to a deficiency in the supply of its stimulus the blood, and resembling that state produced in the lower extremities of the inferior animals, by a ligature of the aorta; or that loss of sensibility in the fingers of man, arising from an obstructed subclavian or humeral artery. *Mr. Wardrop and Mr. Lawrence were inclined, however, to take a very different view of the symptoms, and to ascribe them to a morbid state of the brain itself, arising, in all probability, from an aneurismatic affection of those vessels of the dura mater which, by passing through the cranium, communicate with the superficial arteries of the head, forming, in fact, internally, a part of the disease which was so conspicuous outwardly, and thus by their enlargement exerting a powerful compression on the brain itself. This opinion was highly corroborated by symptoms afterwards noticed, viz. by the great protrusion of the eyeball, and by the circumstance that the thrilling pulsation was most observable in the centre of the tumour, at that point where the cranium appeared almost completely absorbed, and where, in all probability, the freest communication existed with the vessels supplying the membranes of the brain.*"*—*Lancet, 6th October*, 1827.

As there is reason to believe that Mr. Lawrence is not always obliged to a friend for a sight of the LANCET, (as he is for our Journal, of the existence of which he seems to have accidentally heard some time in February, 1828!) we ask him how it happens that he has permitted such "ERRONEOUS ASSERTIONS" to remain uncontradicted in the said LANCET, for more than four months, reserving all the fire of his indignation for us, who only *re-published* the statement?* How is this long silence of Mr. Lawrence to be accounted for? Is it possible that, while poor Nowlan was *alive*, those ingenious physiological and pathological explanations, which we have quoted from Panton Square, were permitted to glide down the stream of time, on the pages of the "INVALUABLE," as coruscations from the mighty intellects of Messrs. Lawrence and Wardrop; but that, four months afterwards, when poor Nowlan's *death and dissection* dissolved, into thin air, these fairy fabricks of the imagination, Mr. Lawrence started, all at once, from his halcyon slumbers—loudly declared that he had never enunciated any thing of the kind—that the whole was an "ABSURDITY"—and an "*erroneous assertion*" by Dr. Johnson!!

Whether Mr. Lawrence may chance to "catch a tartar" in this crusade, remains to be seen. At all events, he has placed upon record, (inadvertently, no doubt,) his own SOLEMN TESTIMONY to the MENDACITY of the LANCET:—and, in aiming a dagger at the veracity of Dr. Johnson, he has plunged that instrument into the bosom of his faithful ally—the LANCET! We are not among those who see the finger of Providence in every instance of moral retribution, in this world; but we will say, that Mr. Lawrence has brought about a piece of "dramatic justice," in this scene, which well deserves to be recorded.

As to Mr. Lawrence's strictures on our *taste* and *feeling*—this is the "*Devil* reproving *Sin*," with a vengeance, after the taste and feeling displayed in "PAUL'S EPISTLES" to his bosom friends—Messrs. Cooper, Travers, and Butter! We leave it to the members of the Medico-Chirurgical Society, who heard Mr. Lawrence's statements, to decide on the correctness or incorrectness of our report, with just as much confidence as Mr. Lawrence does.†

* See our last Fasciculus for the beautiful verification of these views.

* The account of this case is *acknowledged* to be taken from the Lancet, so that Mr. Lawrence can have no pretence for the attempt to father it on us.

† The statement in the Lancet of last week, on this subject, looks a little suspicious of breach of confidence some-

We have given insertion to Mr. Lawrence's letter verbatim, because it is short, and touches on a subject of great intesest in the surgical world—we mean the late operation of Mr. Wardrop. We have, in this, violated a rule which we laid down at the beginning of the year, and given just cause of offence to other correspondents. We have treated Mr. Lawrence with more respect than he can reasonably claim from us, after the supercilious preamble of his letter; but we shall take care, in future, not to tax our readers with altercations between two individuals, for neither of which, perhaps, the public care a straw. The Lancet (which now is absolutely destitute of professional information) is the proper channel for this species of medical literature, and to that publication we shall refer all applicants.

---

### PROSECUTION OF MR. STANLEY.

It is a very general law, we believe, among animals, that individuals of the same species avoid preying on each other. It is true, that many of them are very pugnacious. Cocks will fight while life remains—dogs will worry each other—and bulls will meet with tremendous clash, when enfuriated by animal passions. The history of the human race, unfortunately, proves that nations will unite to exterminate nations, while individuals are perpetually engaged in the ignoble pursuit of supplanting, cheating, scandalizing, and betraying one another! With all these imperfections in poor weak mortals, there has long existed among the liberal and learned professions, a kind of universal compact that men of the same faculty should obey the Christian precept—"do unto others as ye would be done by"—put the most merciful con struction on things—and never attempt to exaggerate their neighbour's failings, or exasperate the passions of the populace against one of their own brethren. This tacit compact has very generally been exemplified in the three learned professions. The recent action, in which Mr. Stanley was amerced in 30 pounds damages, for not discovering the precise nature of an accident, after swelling and inflammation of the parts had come on—and for mistaking a foreign body jammed along-side or beneath the patella, for a portion of that bone broken off, has called forth such a stormy effusion of vituperation from a junto of his brethren, that human nature sickens at the scene! Heaven knows we owe Mr. Stanley no obligation; nor are we the toad-eaters of the Medical Aristocracy to which he is said to belong; but were he our bitterest enemy, we should detest our own existence, if, for one moment, we permitted personal feeling to interfere with the dictates of justice. Mr. Stanley may have committed an error in judgment—errare est humanum—but we do most solemnly protest against the fiat of that *professional* tribunal (if it deserves such a name) which condemns Mr. Stanley, without knowing all the particulars of the case, and merely because an *ignorant jury* gave a verdict in direct opposition to *the judge*, and to several of the most eminent surgeons in London. We have no terms expressive enough of our indignation, at seeing a portion of the medical press take advantage of such a verdict, in order to harrass an individual, who happens to be one of a different party. But Mr. Stanley may console himself with the reflection—nay, with the certainty, that this procedure of the press, will defeat its own illiberal purposes—and that the

---

where—we do not mean on Mr. Lawrence's part. However, we have but one short reply to make:—We defy any member of the Medico-Chirurgical Society to shew that we have ever published "*misstatements*," (the term applied in the Lancet) while reporting the proceedings of that body. Whoever asserts, by letter, by word, or by print, that we have misstated, or misrepresented, any case read, or any opinion given, in that Society, commits himself a "*mis-statement*." Qui capit ille facit.

* If the writers of these inflammatory effusions were acquainted with the prejudices, which medical men have to encounter in their intercourse with the public, they would not be so ready, on all occasions, to increase that prejudice, by holding up the most eminent men in the Profession to ridicule. By this procedure, they put arguments into the mouths of every little tradesman to scoff

Profession, so far from joining in the vulgar outcry, will support him through the momentary struggle. We have said before, that we owe Mr. Stanley no obligation, but we owe our tribute to *justice*, as well as to generosity—and while unmerciful critics point their black artillery at his head, we will publickly and privately espouse his cause—and so we are convinced, will every member of the profession, who has a spark of Christianity, philanthropy, or magnanimity in his breast!

In taking this line, we enter not, at all, into the surgical merits of the case. We have no proper data on which to ground a decision—and, till some circumstantial details of the accident, and the treatment, be laid before the Profession, we will not be so uncharitable as to pass a judgment, much less a censure on the practitioners in attendance.

---

LIBEL—MACLEOD *versus* WAKLEY.

This cause was determined on Monday last, in the Court of KING'S BENCH, before Lord TENTERDEN. The Court was crowded to excess. Sir James Scarlet stated the case to the Jury with great force. The impression on the audience was exceedingly strong, from the intrinsic evidence that nothing was exaggerated—no fact distorted—no expression in the libel coloured. Sir James wound up his charge, by reading, from the Lancet of last Saturday, the prediction, that "the yellow Goth would be *scarified* by Mr. Brougham on Monday." This passage excited strong symptoms of disgust in the countenances of judge, jury, and auditors. Mr. Brougham felt it necessary to apologize for the bad taste of his client, and moved heaven and earth to keep this passage from the face of the record. At length he succeeded by a legal quibble—namely, a denial that there was any proof of Mr. Wakley being editor of the Lancet during the last week! In this he succeeded. But the *moral* effect of the passage was produced beyond redemption.

Mr. Brougham did all that man could do—(but, under the circumstances in which he was placed, his exertions were of little avail)—to persuade the jury, that the false assertions against Dr. Macleod originated in a *mistake*, and that the violent language in which they were conveyed, was a mere *ebullition of feeling* for an injured friend—Mr. WARDROP. But, in this delicate position, Mr. Brougham shewed admirable tact, sound sense, and consummate policy. Instead of complying with the savage anticipation of his client, "the scarification of the yellow Goth," not a single expression was permitted to escape his lips, which could hurt the feelings of the most sensitive mind on earth! On the contrary, he paid Dr. Macleod many handsome compliments, in lieu of the expected scarifications! By this judicious procedure, and by the non attempt of the plaintiff to prove any damages, the defendant was probably saved from a heavy amercement. The verdict was all that could be expected, and five pounds were awarded, which carry the costs of both sides.

The charge of Lord Tenterden (Judge Abbott) will long be remembered by the defendant. Not a single palliating circumstance was mentioned to the jury. The accusations (his Lordship observed) against Dr. Macleod were proved to be unfounded in truth—and if the jury were not convinced of this, they ought to find for the defendant! We suppose a full account of this trial will be published.

Never were judge, jury, and knights of the long robe, so puzzled with carotids, ligatures citra et ultra tumores, post-mortem researches after vanished aneurisms, &c. as on this occasion.

---

at, and insult the GENERAL PRACTITIONER in attendance, whenever he claims a reward for his services. This we see done every day—and this, every practitioner is aware of; but the literary garreteer, who fires off his inflammatory harangues among the public, knows and feels nothing of the matter.

The Lancet boasts of having been the instrument that enlightened the Jury to give damages against Mr. Stanley, in this case, contrary to the opinion of the Judge, and against the evidence of a Cooper, a Brodie, a Bell, a Travers, &c. &c! Be it so! If the Lancet has been able to raise this prejudice in the minds of the "profanum vulgus," against *such men*, it requires but little divination to see that, henceforth, no medical practitioner is one hour safe from prosecution! "These are thy glorious fruits—parent of ill!"

